# Pharmacy Students’ Perceptions and Use of Artificial Intelligence Tools in Oman: A Cross-Sectional Survey

**DOI:** 10.7759/cureus.91757

**Published:** 2025-09-07

**Authors:** Rodaina Ba Makhalef, Noor Al Maashani, Eman I El-Kimary, Raqiya Al Mamari, Abdullah Al Lawati, Hanan Al Lawati

**Affiliations:** 1 Pharmacy Program, Department of Pharmaceutics, Oman College of Health Sciences, Muscat, OMN; 2 College of Medicine and Health Sciences, Sultan Qaboos University, Muscat, OMN

**Keywords:** artificial intelligence in education, artificial intelligence in healthcare, pharmacy students, students’ knowledge, student’s perception

## Abstract

Background: Artificial intelligence (AI) is quickly changing pharmacy education by offering new tools for accessing information, improving research, and supporting academics. In Oman, the use of AI in pharmacy programs is still in the early stages, and there is little data on how students view and interact with these technologies.

Setting and design: This cross-sectional study surveyed pharmacy students from three institutions: the National University of Science and Technology (NU), the University of Nizwa, and Oman College of Health Sciences (OCHS). A custom-designed questionnaire was disseminated via official email platforms and social media. Responses were collected using Microsoft Excel and analyzed with SPSS.

Results: A total of 156 students participated in the survey. The majority were female (n = 108, 69.2%), aged 20-22 years (n = 89, 57.1%), and enrolled in public universities (n = 92, 59.0%). Nearly half (n = 76, 48.7%) reported a moderate understanding of AI, while 34 students (21.8%) reported an advanced understanding. ChatGPT was the most widely used tool (n = 142, 91.0%), followed by Google (n = 104, 66.7%) and Grammarly (n = 59, 37.8%). Despite this high usage, only 15 students (9.6%) had received formal training in AI. Most students, n = 117 (75.0%), believed that AI had a positive effect on classroom learning. While pharmacy students in Oman are actively using AI tools like ChatGPT, 95 students (60.9%) felt comfortable using them to gain knowledge. Common concerns included overreliance (n = 88, 56.4%), reduced creativity (n = 76, 48.7%), and ethical issues (n = 48, 30.8%). Notably, 104 students (66.7%) expressed interest in receiving more AI training or attending workshops.

Conclusion: Pharmacy students in Oman are using AI tools like ChatGPT, but they lack formal training. Educational programs to promote responsible and effective use of AI in pharmacy education are needed.

## Introduction

Artificial intelligence (AI) is a technology that enables computers and machines to simulate human capabilities such as learning, comprehension, problem-solving, decision-making, creativity, and autonomy [1]. Over the last few years, AI has made significant strides across multiple domains, and pharmacy education is no exception. As healthcare and pharmaceutical practices become more complex, there is a growing need to incorporate technological innovations to enhance learning and improve outcomes.

In pharmacy, AI is already influencing areas such as drug discovery, design, delivery, and practice [2]. For example, AI can recognize hit and lead compounds, accelerate drug target validation, and optimize drug structure design [3]. Furthermore, AI-powered clinical decision support systems can provide evidence-based recommendations for drug selection and dosage adjustments, thus improving patient safety and treatment outcomes [4]. As the use of AI continues to expand in pharmacy practice, it is increasingly important to incorporate AI education into pharmacy curricula to ensure that future pharmacists are adequately equipped with the necessary knowledge and skills [5]. The integration of AI in healthcare education has shown promise across various fields. A nationwide multi-center study and a focus group discussion at the University of Tennessee revealed that most medical students believe AI can enhance their educational experience, research skills, and clinical judgment [6,7]. However, both studies also highlight a gap in formal AI training within healthcare curricula [8]. The International Pharmaceutical Federation's 2021 report emphasized a global shortfall in digital health education and recommended the inclusion of AI and related tools in pharmacy programs [9].

Attitudes toward AI in pharmacy education and practice vary. While many students and faculty acknowledge its potential to improve learning, patient care, and medication safety, others remain skeptical - concerned about academic dishonesty, job security, the complexity of AI system implementation, and general unfamiliarity with the technology [10,11]. In addition, ethical issues like data privacy and security are essential, especially since AI systems often depend on sensitive patient data. A 2023 study in the Indian Dermatology Online Journal highlighted concerns about AI-driven diagnostics and the risk of breaking confidentiality if datasets are not properly secured [12,13]. Another key challenge is the lack of technical skills among pharmacy educators. This gap can limit the effective teaching of AI-related topics. Faculty development and training are needed to address this issue. In addition, relying too much on AI may weaken the growth of critical thinking and clinical judgment. Therefore, AI should be integrated as a supportive tool while continuing to emphasize interpersonal communication, empathy, and professional reasoning - skills that are fundamental to effective pharmacy practice and cannot be replaced by machines [13-15].

Despite the global momentum, the integration of AI in pharmacy education remains underexplored in the context of Oman. To our knowledge, this is the first study involving multiple institutions in the country that looks at pharmacy students' perceptions, usage patterns, and readiness for AI in academic settings. This research explores how AI is being adopted, the benefits and drawbacks that people see, and the educational challenges and opportunities it brings. The primary objective was to assess students’ perceptions, usage patterns, and readiness for AI in pharmacy education in Oman. The secondary objectives included identifying perceived benefits, challenges, and institutional support for AI integration.

## Materials and methods

Study design

A cross-sectional study was conducted to assess the role of AI in pharmacy education at Oman College of Health Sciences (OCHS), the National University of Science and Technology (NU), and the University of Nizwa. The questionnaire (see Appendix) was investigator-developed and revised based on feedback regarding clarity and comprehensiveness obtained through meetings with academic supervisors at OCHS. It was then distributed to participants from the three institutions via email and WhatsApp.

Population and sampling

The self-administered questionnaire was distributed among pharmacy students (first, second, third, and fourth years) from OCHS, NU, and the University of Nizwa. The required sample size was determined using the sample size for a population mean formula with finite‑population correction. Parameters were set at a 95% confidence level, 5% margin of error, and an assumed standard deviation based on a conservative 50% response distribution. Using an estimated total population of approximately 600 pharmacy students across the three institutions, the calculated minimum sample size was 235. We obtained 156 complete responses, which corresponds to a margin of error of approximately 6.8% at 95% confidence. Participants included first-, second-, third-, and fourth-year pharmacy students enrolled in pharmacy programs across Oman. Students who had withdrawn from the program or were enrolled in programs from other specialties and fields were excluded from the study. Additionally, a pilot study was conducted prior to the actual study.

Data collection methods

The questionnaire was developed using Google Forms (Google LLC, Mountain View, California, United States) and underwent rigorous review and refinement under the close supervision of experienced academic faculty with expertise in pharmacy education, research methodology, and digital health. These supervisors provided critical input to ensure that the phrasing, structure, and content of the questionnaire met high academic and ethical standards, maximizing clarity, relevance, and content validity. The development process was informed by a review of existing literature on AI in pharmacy education and aligned with current trends and gaps identified in recent studies, forming the scientific basis for questionnaire design. The final tool was designed to be both comprehensive and context-sensitive, comprising two main sections. The first section gathered key demographic information, including age, gender, institution name and sector, and year of study. The second section focused on participants’ knowledge and perceptions of AI in pharmacy education. It included a combination of Likert-scale items (1 to 5) assessing dimensions such as familiarity, comfort, encouragement, and self-rated proficiency, as well as a range of open-ended, binary (yes/no), and multiple-choice questions to enrich the data.

Data collection spanned three months, and participation was entirely voluntary. Reminders were sent via email and social media platforms once every two weeks to enhance response rates. Responses were collected electronically and compiled in Microsoft Excel, then analyzed using IBM SPSS Statistics for Windows, Version 27.0 (released 2019, IBM Corp., Armonk, NY). Continuous variables were presented as means and standard deviations (SD) or medians and interquartile ranges (IQR), based on their distribution, while categorical variables were summarized using frequencies and percentages. Response bias was minimized by ensuring anonymity and clear instructions. Cases with incomplete or missing data were excluded from analysis. The analysis provided a detailed descriptive overview of participant demographics, patterns of AI tool usage, and the perceived impact of AI on pharmacy education.

Ethical consideration

Prior to data collection, ethical approval was obtained from the OCHS Research and Ethics Committee, Sultanate of Oman, on October 23, 2024. The approval code is Ref. No. OCHS/REC/PROPSAL-APPROVED/45/2024.

## Results

Demographic characteristics

The survey gathered responses from 156 participants across different universities and colleges in Oman. It explored the sector of the institutions attended - whether public or private. Most participants were enrolled at public universities (n = 92, 59.0%), while 64 (41.0%) attended private institutions. A substantial portion of the sample came from OCHS (n = 92, 59.0%), followed by the University of Nizwa (n = 48, 30.8%) and NU (n = 16, 10.2%).

In terms of age, the majority of participants (n = 89, 57.0%) fell within the 20-22 age range. The 23-25 age group represented 33 students (21.1%), while the 17-19 age group accounted for 19 students (12.2%). Only 15 participants (9.7%) were above 26 years old. Most responses were from females (n = 108, 69.2%), while 48 (30.8%) were male. The largest group of participants was fourth-year students (n = 53, 34.0%), followed by third-year students (n = 41, 26.3%). First-year (n = 24, 15.4%) and second-year students (n = 22, 14.1%) formed a smaller portion, while 16 participants (10.2%) were graduates (*Other* category). Table 1 displays the demographic characteristics of the participants.

**Table 1:** Demographic characteristics of the participants (n = 156)

Variable	Category	n (%)
Age	17–19	19 (12.2%)
	20–22	89 (57.0%)
	23–25	33 (21.1%)
	>26	15 (9.7%)
Gender	Female	108 (69.2%)
	Male	48 (30.8%)
Year of study	First year	24 (15.4%)
	Second year	22 (14.1%)
	Third year	41 (26.3%)
	Fourth year	53 (34.0%)
	Other (Graduates)	16 (10.2%)
Sector of the university	Public	92 (59.0%)
	Private	64 (41.0%)
University/college name	Oman College of Health Sciences (OCHS)	92 (59.0%)
	University of Nizwa	48 (30.8%)
	National University of Science and Technology (NU)	16 (10.2%)

Knowledge levels of AI

**Figure 1:**
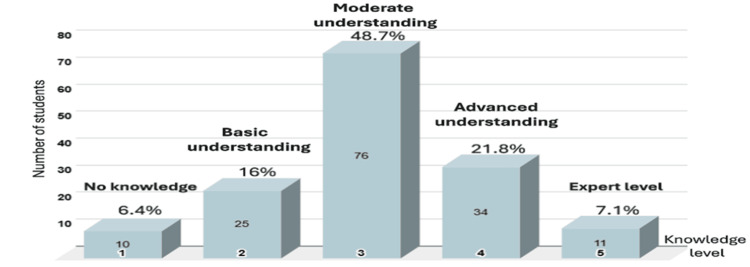
Knowledge level of artificial intelligence (AI) (n = 156) Knowledge levels were rated on a five-point scale, where 1 = very little or no knowledge, 2 = basic understanding, 3 = moderate understanding, 4 = advanced understanding, and 5 = expert-level knowledge.

Regarding the knowledge levels of AI, as shown in Figure 1, most respondents (n = 76, 48.7%) reported a moderate understanding. A notable portion (n = 34, 21.8%) had an advanced understanding, while 11 students (7.1%) identified as having expert-level knowledge. A total of 25 students (16.0%) rated their knowledge as basic, and 10 students (6.4%) reported very little or no knowledge. The overall mean AI knowledge score was 3.05 out of 5, reflecting a moderate level of understanding across the sample.

ChatGPT was the most used AI tool (n = 142, 91.0%), followed by Google (n = 104, 66.7%) and Grammarly (n = 59, 37.8%). Other tools were used at lower rates, including Bites (n = 11, 7.1%), Deepseek (n = 5, 3.4%), Copilot (n = 4, 2.8%), and others. Most students (n = 130, 83.3%) reported not using citation tools such as EndNote, Mendeley, or Zotero.

Purpose of AI use

Regarding the purpose of AI use, 83 students (53.2%) used it for a combination of reasons. Specifically, 26 students (16.7%) used AI for clarifying doubts and 26 (16.7%) for research. Others used it for assessments (12 students, 7.7%), reading slides (six students, 3.8%), and summarizing (one student, 0.6%), while two students (1.3%) did not use AI for any listed purposes. In total, 95 students (60.9%) agreed with the idea of using AI to acquire knowledge, reflecting an overall positive attitude toward AI-assisted learning.

Most respondents (56 students, 35.9%) felt comfortable using AI to acquire knowledge, 39 (25.0%) were very comfortable, 46 (29.5%) were neutral, 15 (9.6%) felt uncomfortable, and two (1.3%) were very uncomfortable. In total, 117 students (75.0%) believed AI positively impacted classroom activities, and 155 (99.4%) agreed that it helped them access information.

Institutional support for AI integration

When asked whether their college encourages AI use, 57 students (36.5%) were neutral, 51 (32.7%) felt encouragement was slight, and 17 (10.9%) believed there was no encouragement. Only 25 students (16.0%) felt encouraged, and six (3.8%) felt strongly encouraged. Overall, 31 students (19.8%) reported feeling either encouraged or strongly encouraged to use AI in their studies.

**Figure 2:**
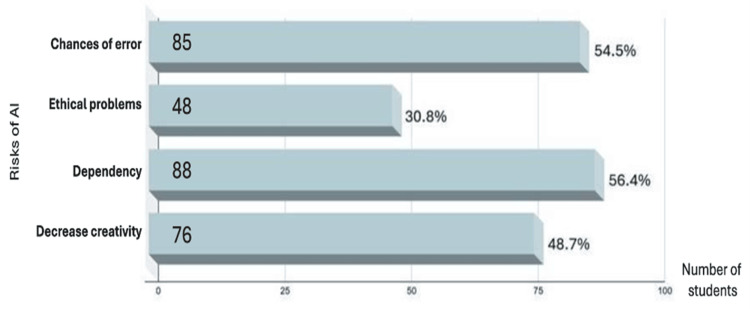
Perspectives on the risks of using artificial intelligence (AI) in pharmacy education

Regarding teachers' use of AI in preparing lectures or materials, 73 students (46.8%) said yes, while 83 (53.2%) said no. A majority of students (134, 85.9%) believed that AI should be included in the pharmacy curriculum, while 22 (14.1%) did not. On risks, as shown in Figure 2, the most common concern was dependency on AI (88 students, 56.4%), followed by chances of error (85, 54.5%), reduced creativity (76, 48.7%), and ethical issues (48, 30.8%). As for reasons for using advanced technologies, 105 students (67.3%) said that it made information more accessible. Others cited time management (42, 26.9%), reduced costs (six, 3.8%), and a small group (three, 1.9%) reported all the above. Finally, 104 students (66.7%) believed that AI training or workshops were needed, while 52 (33.3%) did not. Only 15 students (9.6%) had received any formal training.

## Discussion

The use of AI in pharmacy education provides students with a chance to improve their learning experience [16]. This makes it vital for pharmacy students to understand AI. In our study, the participants showed a moderate understanding of AI, with an average score of 3.05 out of 5. This indicates that most students know about AI concepts, but they need to engage more deeply and learn more advanced topics. This finding agrees with an earlier multinational study, which found that pharmacy students had a moderate level of AI knowledge, scoring 42.3 ± 21.8 out of 100 [17]. Another study conducted in Saudi Arabia indicated that pharmacy students had good knowledge of AI [18].

AI tools are gaining popularity in education due to their ability to support learning and enhance understanding. In our study, 95 students (60.9%) agreed with the idea of using AI to acquire knowledge. Tools like ChatGPT operate using complex machine-learning algorithms that compare user input to large datasets [19]. These algorithms process and learn from data patterns in steps. This allows AI to generate relevant and coherent responses [20]. Although this makes AI tools a useful source of information, they should be used carefully. In an international study, pharmacy students saw AI as a helpful second opinion in medical contexts [5]. Interestingly, when ChatGPT was asked, "What do you think of using you to acquire knowledge?" it responded: "I am a super smart assistant, but not a replacement for human-based judgment," emphasizing the importance of verifying AI-generated information through critical thinking.

Among the various AI platforms available, such as Claude, Gemini, DeepSeek, and Grok, ChatGPT emerged as the most commonly used tool in our study, with 142 students (91.0%) reporting its use. This usage rate is notably higher than findings from other studies, where approximately half of the students had used ChatGPT [21]. The tool’s popularity can be attributed to its adaptability, ease of use, human-like responses, and continuous development within an open-source and ethically aware ecosystem [22].

Despite the growing use of AI, our study revealed that 130 students (83.3%) had not used citation tools such as EndNote, Mendeley, or Zotero. These tools offer valuable features. EndNote is useful for fields that need structured citation styles. Mendeley works well for research with many PDFs. Zotero helps capture various online content in an open-source format [23]. Their limited use among students might suggest a lack of awareness, formal training, or integration into academic programs. Female students made up the majority of our sample (n = 108, 69.2%), which may reflect either a general trend of higher female enrollment in pharmacy programs or a greater willingness to participate in surveys. This gender distribution is consistent with a multinational study in which 70.3% of respondents were female, reinforcing the observed dominance of females in the pharmaceutical education sector [17].

Although AI has clear value in pharmacy education, our findings highlight the lack of formal instruction or institutional support for its use. Only 31 students (19.8%) felt their college encouraged AI adoption, and just 15 students (9.6%) had received official training. This is consistent with findings by Syed et al. (2023), who reported that 80% of senior pharmacy students in Saudi Arabia had not received any formal AI training [18]. This gap shows the urgent need to create a curriculum, train faculty, and start initiatives at the institutional level. These actions will help prepare future pharmacists with the skills needed to work in the changing world of AI in healthcare.

Limitations

This study faced some limitations, most notably a lower-than-target response rate. Although the calculated sample size was 235, responses were received from 156 students, yielding a response rate of 66.4%. This is within an acceptable range for academic surveys and still provides valuable insights [24]. However, the unachieved target sample size may limit the generalizability of the findings. The inclusion of students from three diverse institutions, covering both public and private sectors, adds strength to the generalizability of the findings. Given the cross-sectional nature of the study, causal relationships between variables cannot be established. However, future studies involving larger and more evenly distributed samples may help validate these observations further. In addition, no inferential statistical analyses were performed, which may have limited the ability to identify significant associations between participant characteristics and AI-related perceptions or behaviors.

## Conclusions

The study examined pharmacy students' knowledge, use, and views on AI. The average AI knowledge among the participants was moderate, scoring 3.05 out of 5. ChatGPT was the most used tool. Even with AI's increasing presence, only 9.6% of students had formal training, showing a significant gap. While AI holds substantial potential to enhance learning in pharmacy education, it also presents notable risks that must be carefully addressed to ensure its responsible and effective implementation. Based on these findings, we recommend the structured integration of AI literacy modules and hands-on workshops into undergraduate pharmacy curricula in Oman to promote responsible, ethical, and skill-based use of AI tools.

## Appendices

AI survey questionnaire

Section A. Demographics

Age ☐ 17-19 ☐ 20-22 ☐ 23-25 ☐ >26

Gender ☐ Female ☐ Male

Year of study ☐ First year ☐ Second year ☐ Third year ☐ Fourth year ☐ Other: ______

Sector of your university ☐ Public ☐ Private

University/college name ☐ Oman College of Health Sciences (OCHS) ☐ University of Nizwa ☐ National University ☐ Other: ______
 

Section B. Knowledge, Use, and Attitudes Toward AI

How do you rate your knowledge level of AI? (1=Very low, 5=Very high) ☐ 1 ☐ 2 ☐ 3 ☐ 4 ☐ 5

Which AI tools are you currently using? (Select all that apply) ☐ ChatGPT ☐ Google ☐ Grammarly ☐ Wolfram Alpha ☐ Bites ☐ Other: ______

What is your purpose for using AI? (Select all that apply) ☐ Clarify doubts ☐ Reading materials/slides ☐ For assessments ☐ For research ☐ All of the above ☐ None ☐ Other: ______

How comfortable are you with the idea of using AI to gain knowledge? (1=Not at all comfortable, 5=Very comfortable) ☐ 1 ☐ 2 ☐ 3 ☐ 4 ☐ 5

Do you think your college encourages students to use technology and AI in academics? (1=Strongly disagree, 5=Strongly agree) ☐ 1 ☐ 2 ☐ 3 ☐ 4 ☐ 5

Has any of your teachers revealed the use of AI in preparing lectures or study materials? ☐ Yes ☐ No

Do you think it is important to integrate AI into the pharmacy curriculum? ☐ Yes ☐ No

What are the risks of using AI in pharmacy education? (Select all that apply) ☐ Chances of error ☐ Ethical problems ☐ Dependency ☐ Decreased creativity

Why do you use advanced technologies? ☐ Decrease cost ☐ Easy access to information ☐ Time management ☐ All of the above

Do you think you need to attend workshops/training sessions about AI? ☐ Yes ☐ No

Have you received any proper/official/formal training related to AI? ☐ Yes ☐ No

If yes, please specify the type of training: ______

Has the use of AI in your classroom activities had an impact on your skills or skill development? ☐ Yes ☐ No

Have you used any of the following tools for citation in your academic work? ☐ EndNote ☐ Mendeley ☐ Zotero ☐ None ☐ Other: ______

Do you agree that AI helps students access information more easily? ☐ Yes ☐ No

Scoring and Data Handling

Likert-scale items (Q6, Q9, Q10): Scored from 1 to 5. For knowledge (Q6), scores were categorized as: 1-1.5 = very little/none, 1.6-2.5 = basic, 2.6-3.5 = moderate, 3.6-4.5 = advanced, 4.6-5 = expert. Mean values were also reported.

Multiple-choice questions (Q7, Q8, Q13, Q14, Q19): Options were treated as multiple-response sets. Results are reported as counts and percentages of respondents selecting each option.

Yes/No questions (Q11, Q12, Q15, Q16, Q18, Q20): Reported as proportions (%).

Open-ended (Q17): Responses were thematically analyzed. Themes were summarized descriptively, with representative examples included in the Results.
